# (*E*,*E*)-*N* ^1^,*N* ^4^-Bis(2,6-difluoro­benzyl­idene)butane-1,4-diamine

**DOI:** 10.1107/S1600536811044801

**Published:** 2011-10-29

**Authors:** Mohammad Khaledi Sardashti, Reza Kia, William Clegg, Ross W. Harrington

**Affiliations:** a Department of Chemistry, Faculty of Science, Islamic Azad University, Shahrekord Branch, Box 166, Tehran, Iran; b Department of Chemistry, Science and Research Branch, Islamic Azad University, Tehran, Iran; c School of Chemistry, Newcastle University, Newcastle upon Tyne NE1 7RU, England

## Abstract

The asymmetric unit of the title compound, C_18_H_16_F_4_N_2_, comprises two half crystallographically independent potentially bidentate Schiff base ligands, with an inversion centre located at the mid-point of the central C—C bond. The crystal packing is stabilized by inter­molecular C—H⋯F and π–π inter­actions [centroid–centroid distance = 3.8283 (11) Å].

## Related literature

For background to the synthesis and structural variations of Schiff base ligands and their complexes, see: Granovski *et al.* (1993 ▶); Elmali *et al.* (2000 ▶).
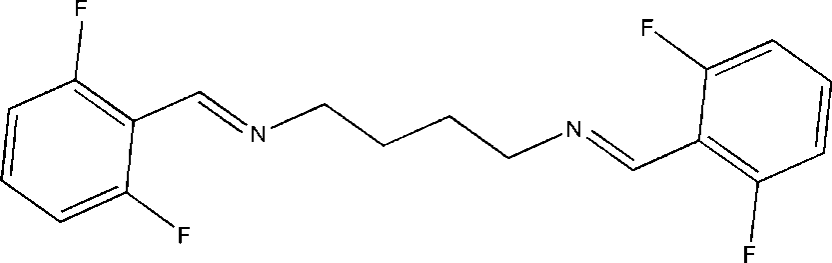


## Experimental

### 

#### Crystal data

C_18_H_16_F_4_N_2_
*M* *_r_* = 336.33
Triclinic, 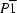

*a* = 6.4672 (8) Å
*b* = 8.9296 (12) Å
*c* = 14.4939 (19) Å
α = 104.956 (2)°
β = 94.474 (2)°
γ = 93.679 (2)°
*V* = 803.10 (18) Å^3^
*Z* = 2
Mo *K*α radiation
μ = 0.12 mm^−1^
*T* = 150 K
0.34 × 0.30 × 0.20 mm

#### Data collection

Bruker SMART 1K CCD area-detector diffractometer
Absorption correction: multi-scan (*SADABS*; Bruker, 2005 ▶) *T*_min_ = 0.962, *T*_max_ = 0.977
5828 measured reflections
2819 independent reflections
2394 reflections with *I* > 2σ(*I*)
*R*_int_ = 0.019

#### Refinement

*R*[*F* ^2^ > 2σ(*F* ^2^)] = 0.037
*wR*(*F* ^2^) = 0.100
*S* = 1.11
2819 reflections
218 parameters
H-atom parameters constrained
Δρ_max_ = 0.25 e Å^−3^
Δρ_min_ = −0.17 e Å^−3^

### 

Data collection: *SMART* (Bruker, 2005 ▶); cell refinement: *SAINT* (Bruker, 2005 ▶); data reduction: *SAINT*; program(s) used to solve structure: *SHELXS97* (Sheldrick, 2008 ▶); program(s) used to refine structure: *SHELXL97* (Sheldrick, 2008 ▶); molecular graphics: *SHELXTL* (Sheldrick, 2008 ▶); software used to prepare material for publication: *SHELXTL* and *PLATON* (Spek,2009 ▶).

## Supplementary Material

Crystal structure: contains datablock(s) I, global. DOI: 10.1107/S1600536811044801/su2336sup1.cif

Structure factors: contains datablock(s) I. DOI: 10.1107/S1600536811044801/su2336Isup2.hkl

Supplementary material file. DOI: 10.1107/S1600536811044801/su2336Isup3.cml

Additional supplementary materials:  crystallographic information; 3D view; checkCIF report

## Figures and Tables

**Table 1:** Hydrogen-bond geometry (Å, °)

*D*—H⋯*A*	*D*—H	H⋯*A*	*D*⋯*A*	*D*—H⋯*A*
C3—H3⋯F3^i^	0.95	2.43	3.137 (2)	131
C7—H7⋯F1^ii^	0.95	2.54	3.378 (2)	148
C12—H12⋯F2	0.95	2.42	3.192 (2)	138

Symmetry codes: (i) 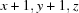; (ii) 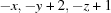.

**Fig. 1.**
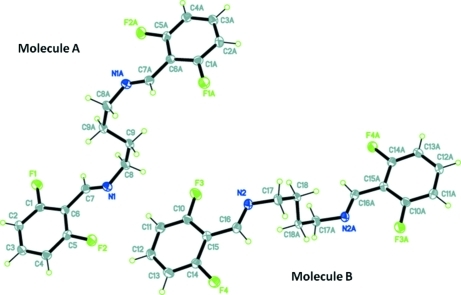
The molecular structure of the two indpendent molecules of the title compound, showing 50% probability displacement ellipsoids and the atomic numbering scheme [Symmetry codes for suffix A: -x, -y + 1, -z + 1 for molecule A, and -x, -y, -z + 2 for molecule B].

**Fig. 2.**
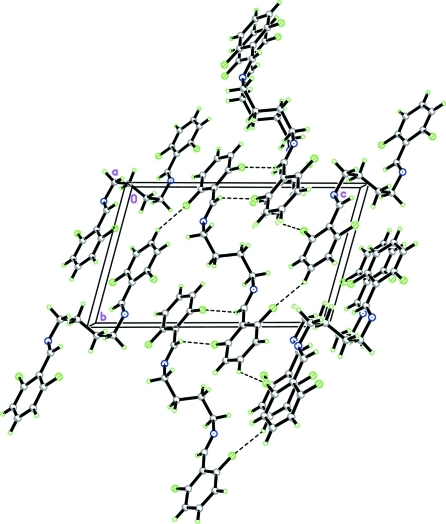
The crystal packing, viewed down the a axis, showing the intermolecular C—H···F hydrogen bonds (dashed lines), which link the molecules to form a three-dimensional network.

## supplementary crystallographic information

### Comment

Schiff base ligands are among the most prevalent ligands in the field of coordination chemistry. Metal derivatives of Schiff bases have been studied extensively, and Ni^II^ and Cu^II^ complexes play a major role in both synthetic and structural research (Elmali *et al.*, 2000; Granovski *et al.*, 1993).

The asymmetric unit of the title compound comprises two half crystallographically independent Schiff base molecules; A (including N1) and B (including N2), see Fig. 1. Each molecule lies about an inversion centre, which is located in the middle of the central C—C bond (Fig. 1). In both molecules the aromatic ring and the imine segment (C—N═C) are approximately coplanar [dihedral angle 14.9 (2)° for molecule A and 3.4 (2)° for molecule B]. These two essentially planar units are linked by a step formed by the four CH_2_ groups, so that they are strictly parallel by inversion symmetry but not coplanar.

The crystal packing is stabilized by intermolecular C—H···F interactions (Fig. 2 and Table 1), and by ring stacking of the benzene rings of the two independent molecules [centroid···centroid distance 3.8283 (11) Å, dihedral angle 2.33 (8)°].

### Experimental

The title compound was synthesized by mixing 2,4-difluorobenzaldehyde (4 mmol) and butylenediamine (2 mmol) in chloroform (20 ml). After stirring for 2 h, the solution was filtered and the resulting yellow solid was crystallized from ethanol, giving single crystals suitable for X-ray diffraction.

### Refinement

All H atoms were positioned geometrically and constrained to ride on the parent atoms: C-H = 0.95 and 0.99 %A for CH and CH_2_ H atoms, respectively, with U_iso_(H) = 1.2 *U*_eq_(C).

### Crystal data

**Table:**

C_18_H_16_F_4_N_2_	*Z* = 2
*M**_r_* = 336.33	*F*(000) = 348
Triclinic, *P*1	*D*_x_ = 1.391 Mg m^−^^3^
Hall symbol: -P 1	Mo *K*α radiation, λ = 0.71073 Å
*a* = 6.4672 (8) Å	Cell parameters from 4637 reflections
*b* = 8.9296 (12) Å	θ = 2.9–28.3°
*c* = 14.4939 (19) Å	µ = 0.12 mm^−^^1^
α = 104.956 (2)°	*T* = 150 K
β = 94.474 (2)°	Block, colourless
γ = 93.679 (2)°	0.34 × 0.30 × 0.20 mm
*V* = 803.10 (18) Å^3^	

### Data collection

**Table:**

Bruker SMART 1K CCD area-detector diffractometer	2819 independent reflections
Radiation source: fine-focus sealed tube	2394 reflections with *I* > 2σ(*I*)
graphite	*R*_int_ = 0.019
Detector resolution: 8.33 pixels mm^-1^	θ_max_ = 25.0°, θ_min_ = 2.4°
ω scans	*h* = −7→7
Absorption correction: multi-scan (*SADABS*; Bruker, 2005)	*k* = −10→10
*T*_min_ = 0.962, *T*_max_ = 0.977	*l* = −16→17
5828 measured reflections	

### Refinement

**Table:**

Refinement on *F*^2^	Secondary atom site location: difference Fourier map
Least-squares matrix: full	Hydrogen site location: inferred from neighbouring sites
*R*[*F*^2^ > 2σ(*F*^2^)] = 0.037	H-atom parameters constrained
*wR*(*F*^2^) = 0.100	*w* = 1/[σ^2^(*F*_o_^2^) + (0.0437*P*)^2^ + 0.2616*P*] where *P* = (*F*_o_^2^ + 2*F*_c_^2^)/3
*S* = 1.11	(Δ/σ)_max_ < 0.001
2819 reflections	Δρ_max_ = 0.25 e Å^−^^3^
218 parameters	Δρ_min_ = −0.17 e Å^−^^3^
0 restraints	Extinction correction: *SHELXL97* (Sheldrick, 2008), Fc^*^=kFc[1+0.001xFc^2^λ^3^/sin(2θ)]^-1/4^
Primary atom site location: structure-invariant direct methods	Extinction coefficient: 0.0064 (19)

### Special details

**Table:**

Experimental. The low-temperature data were collected with the Oxford Cyrosystems Cryostream low-temperature attachment.
Geometry. All e.s.d.'s (except the e.s.d. in the dihedral angle between two l.s. planes) are estimated using the full covariance matrix. The cell e.s.d.'s are taken into account individually in the estimation of e.s.d.'s in distances, angles and torsion angles; correlations between e.s.d.'s in cell parameters are only used when they are defined by crystal symmetry. An approximate (isotropic) treatment of cell e.s.d.'s is used for estimating e.s.d.'s involving l.s. planes.
Refinement. Refinement of *F*^2^ against ALL reflections. The weighted *R*-factor *wR* and goodness of fit *S* are based on *F*^2^, conventional *R*-factors *R* are based on *F*, with *F* set to zero for negative *F*^2^. The threshold expression of *F*^2^ > σ(*F*^2^) is used only for calculating *R*-factors(gt) *etc*. and is not relevant to the choice of reflections for refinement. *R*-factors based on *F*^2^ are statistically about twice as large as those based on *F*, and *R*- factors based on ALL data will be even larger.

### Fractional atomic coordinates and isotropic or equivalent isotropic displacement parameters (Å^2^)

**Table:**

	*x*	*y*	*z*	*U*_iso_*/*U*_eq_	
N1	0.1808 (2)	0.73556 (16)	0.64201 (10)	0.0304 (3)	
N2	0.1964 (2)	0.08666 (15)	0.86448 (9)	0.0280 (3)	
F1	0.19530 (16)	1.11574 (12)	0.54522 (8)	0.0423 (3)	
F2	0.57474 (16)	0.85519 (11)	0.73666 (7)	0.0392 (3)	
F3	0.14208 (16)	0.33304 (13)	0.78309 (9)	0.0486 (3)	
F4	0.76402 (16)	0.30222 (12)	0.96638 (8)	0.0424 (3)	
C1	0.3727 (2)	1.10083 (19)	0.59734 (11)	0.0274 (4)	
C2	0.5378 (3)	1.21002 (19)	0.60645 (12)	0.0327 (4)	
H2	0.5284	1.2924	0.5764	0.039*	
C3	0.7184 (3)	1.1967 (2)	0.66064 (12)	0.0323 (4)	
H3	0.8348	1.2707	0.6677	0.039*	
C4	0.7309 (2)	1.07689 (19)	0.70447 (12)	0.0290 (4)	
H4	0.8539	1.0686	0.7425	0.035*	
C5	0.5610 (2)	0.96960 (18)	0.69179 (11)	0.0260 (4)	
C6	0.3742 (2)	0.97517 (17)	0.63782 (10)	0.0236 (3)	
C7	0.1858 (2)	0.86668 (18)	0.62385 (11)	0.0262 (4)	
H7	0.0593	0.8975	0.5995	0.031*	
C8	−0.0223 (3)	0.6452 (2)	0.62406 (12)	0.0353 (4)	
H8A	−0.1286	0.7057	0.6011	0.042*	
H8B	−0.0627	0.6257	0.6847	0.042*	
C9	−0.0163 (3)	0.49050 (19)	0.54960 (12)	0.0324 (4)	
H9A	0.0978	0.4343	0.5706	0.039*	
H9B	−0.1488	0.4263	0.5459	0.039*	
C10	0.3370 (2)	0.38982 (19)	0.82192 (12)	0.0285 (4)	
C11	0.4160 (3)	0.52544 (19)	0.80426 (12)	0.0335 (4)	
H11	0.3351	0.5768	0.7664	0.040*	
C12	0.6160 (3)	0.58532 (19)	0.84294 (12)	0.0336 (4)	
H12	0.6728	0.6790	0.8316	0.040*	
C13	0.7339 (3)	0.51022 (19)	0.89783 (12)	0.0331 (4)	
H13	0.8712	0.5509	0.9245	0.040*	
C14	0.6470 (3)	0.37536 (18)	0.91265 (12)	0.0275 (4)	
C15	0.4472 (2)	0.30737 (17)	0.87626 (11)	0.0245 (3)	
C16	0.3732 (2)	0.15886 (18)	0.89413 (11)	0.0261 (4)	
H16	0.4662	0.1137	0.9310	0.031*	
C17	0.1558 (3)	−0.06100 (18)	0.88829 (12)	0.0298 (4)	
H17A	0.2825	−0.0846	0.9229	0.036*	
H17B	0.1225	−0.1454	0.8284	0.036*	
C18	−0.0243 (2)	−0.05570 (17)	0.95036 (11)	0.0248 (3)	
H18A	−0.1474	−0.0238	0.9176	0.030*	
H18B	−0.0605	−0.1615	0.9571	0.030*	

### Atomic displacement parameters (Å^2^)

**Table:**

	*U*^11^	*U*^22^	*U*^33^	*U*^12^	*U*^13^	*U*^23^
N1	0.0337 (8)	0.0262 (7)	0.0285 (7)	−0.0040 (6)	0.0007 (6)	0.0044 (6)
N2	0.0307 (8)	0.0286 (7)	0.0266 (7)	−0.0016 (6)	0.0052 (6)	0.0109 (6)
F1	0.0344 (6)	0.0431 (6)	0.0530 (7)	−0.0030 (5)	−0.0134 (5)	0.0260 (5)
F2	0.0374 (6)	0.0374 (6)	0.0489 (6)	0.0016 (4)	−0.0052 (5)	0.0257 (5)
F3	0.0304 (6)	0.0456 (6)	0.0743 (8)	−0.0077 (5)	−0.0171 (5)	0.0333 (6)
F4	0.0352 (6)	0.0387 (6)	0.0568 (7)	−0.0022 (4)	−0.0143 (5)	0.0255 (5)
C1	0.0260 (8)	0.0301 (8)	0.0258 (8)	0.0033 (7)	−0.0027 (6)	0.0084 (7)
C2	0.0371 (10)	0.0287 (9)	0.0350 (9)	−0.0022 (7)	0.0009 (7)	0.0155 (7)
C3	0.0289 (9)	0.0348 (9)	0.0318 (9)	−0.0058 (7)	0.0035 (7)	0.0081 (7)
C4	0.0230 (8)	0.0350 (9)	0.0283 (8)	0.0023 (7)	0.0009 (7)	0.0075 (7)
C5	0.0308 (9)	0.0254 (8)	0.0241 (8)	0.0054 (7)	0.0048 (7)	0.0092 (6)
C6	0.0265 (8)	0.0231 (8)	0.0200 (7)	0.0018 (6)	0.0043 (6)	0.0029 (6)
C7	0.0260 (8)	0.0274 (8)	0.0242 (8)	0.0016 (6)	0.0019 (6)	0.0053 (6)
C8	0.0376 (10)	0.0330 (9)	0.0333 (9)	−0.0093 (8)	0.0081 (8)	0.0069 (7)
C9	0.0382 (10)	0.0260 (8)	0.0323 (9)	−0.0096 (7)	0.0027 (7)	0.0098 (7)
C10	0.0240 (8)	0.0277 (8)	0.0334 (9)	0.0004 (7)	−0.0009 (7)	0.0088 (7)
C11	0.0392 (10)	0.0287 (9)	0.0349 (9)	0.0038 (7)	−0.0034 (8)	0.0142 (7)
C12	0.0421 (10)	0.0228 (8)	0.0365 (9)	−0.0041 (7)	0.0004 (8)	0.0116 (7)
C13	0.0301 (9)	0.0285 (9)	0.0382 (10)	−0.0053 (7)	−0.0045 (7)	0.0087 (7)
C14	0.0278 (9)	0.0261 (8)	0.0297 (8)	0.0048 (7)	−0.0007 (7)	0.0100 (7)
C15	0.0262 (8)	0.0229 (8)	0.0250 (8)	0.0031 (6)	0.0055 (6)	0.0062 (6)
C16	0.0271 (9)	0.0269 (8)	0.0269 (8)	0.0058 (7)	0.0049 (7)	0.0101 (7)
C17	0.0370 (9)	0.0243 (8)	0.0284 (9)	−0.0009 (7)	0.0064 (7)	0.0076 (7)
C18	0.0281 (8)	0.0212 (7)	0.0246 (8)	−0.0053 (6)	−0.0003 (6)	0.0080 (6)

### Geometric parameters (Å, °)

**Table:**

N1—C7	1.264 (2)	C8—H8B	0.990
N1—C8	1.465 (2)	C9—C9^i^	1.520 (3)
N2—C16	1.262 (2)	C9—H9A	0.990
N2—C17	1.460 (2)	C9—H9B	0.990
F1—C1	1.3568 (18)	C10—C11	1.376 (2)
F2—C5	1.3492 (18)	C10—C15	1.396 (2)
F3—C10	1.3504 (19)	C11—C12	1.384 (2)
F4—C14	1.3562 (18)	C11—H11	0.950
C1—C2	1.373 (2)	C12—C13	1.382 (2)
C1—C6	1.394 (2)	C12—H12	0.950
C2—C3	1.385 (2)	C13—C14	1.370 (2)
C2—H2	0.950	C13—H13	0.950
C3—C4	1.382 (2)	C14—C15	1.394 (2)
C3—H3	0.950	C15—C16	1.473 (2)
C4—C5	1.379 (2)	C16—H16	0.950
C4—H4	0.950	C17—C18	1.522 (2)
C5—C6	1.398 (2)	C17—H17A	0.990
C6—C7	1.474 (2)	C17—H17B	0.990
C7—H7	0.950	C18—C18^ii^	1.520 (3)
C8—C9	1.523 (2)	C18—H18A	0.990
C8—H8A	0.990	C18—H18B	0.990
			
C7—N1—C8	116.31 (15)	H9A—C9—H9B	107.8
C16—N2—C17	116.69 (14)	F3—C10—C11	117.55 (14)
F1—C1—C2	117.90 (14)	F3—C10—C15	118.52 (14)
F1—C1—C6	117.45 (14)	C11—C10—C15	123.92 (15)
C2—C1—C6	124.65 (15)	C10—C11—C12	118.54 (15)
C1—C2—C3	118.21 (15)	C10—C11—H11	120.7
C1—C2—H2	120.9	C12—C11—H11	120.7
C3—C2—H2	120.9	C13—C12—C11	120.70 (15)
C4—C3—C2	120.62 (16)	C13—C12—H12	119.7
C4—C3—H3	119.7	C11—C12—H12	119.7
C2—C3—H3	119.7	C14—C13—C12	118.07 (16)
C5—C4—C3	118.57 (15)	C14—C13—H13	121.0
C5—C4—H4	120.7	C12—C13—H13	121.0
C3—C4—H4	120.7	F4—C14—C13	117.68 (15)
F2—C5—C4	117.69 (14)	F4—C14—C15	117.52 (14)
F2—C5—C6	118.30 (14)	C13—C14—C15	124.80 (15)
C4—C5—C6	123.98 (15)	C14—C15—C10	113.96 (14)
C1—C6—C5	113.95 (14)	C14—C15—C16	119.93 (14)
C1—C6—C7	119.55 (14)	C10—C15—C16	126.07 (15)
C5—C6—C7	126.46 (14)	N2—C16—C15	125.39 (15)
N1—C7—C6	124.53 (15)	N2—C16—H16	117.3
N1—C7—H7	117.7	C15—C16—H16	117.3
C6—C7—H7	117.7	N2—C17—C18	111.32 (13)
N1—C8—C9	111.07 (14)	N2—C17—H17A	109.4
N1—C8—H8A	109.4	C18—C17—H17A	109.4
C9—C8—H8A	109.4	N2—C17—H17B	109.4
N1—C8—H8B	109.4	C18—C17—H17B	109.4
C9—C8—H8B	109.4	H17A—C17—H17B	108.0
H8A—C8—H8B	108.0	C18^ii^—C18—C17	113.13 (16)
C9^i^—C9—C8	112.91 (17)	C18^ii^—C18—H18A	109.0
C9^i^—C9—H9A	109.0	C17—C18—H18A	109.0
C8—C9—H9A	109.0	C18^ii^—C18—H18B	109.0
C9^i^—C9—H9B	109.0	C17—C18—H18B	109.0
C8—C9—H9B	109.0	H18A—C18—H18B	107.8
			
F1—C1—C2—C3	179.14 (15)	F3—C10—C11—C12	−179.66 (15)
C6—C1—C2—C3	−0.9 (3)	C15—C10—C11—C12	−0.5 (3)
C1—C2—C3—C4	−0.2 (3)	C10—C11—C12—C13	0.2 (3)
C2—C3—C4—C5	1.0 (2)	C11—C12—C13—C14	0.0 (3)
C3—C4—C5—F2	−178.75 (14)	C12—C13—C14—F4	179.83 (15)
C3—C4—C5—C6	−0.8 (2)	C12—C13—C14—C15	0.1 (3)
F1—C1—C6—C5	−178.90 (13)	F4—C14—C15—C10	179.92 (14)
C2—C1—C6—C5	1.2 (2)	C13—C14—C15—C10	−0.3 (2)
F1—C1—C6—C7	−0.8 (2)	F4—C14—C15—C16	−2.0 (2)
C2—C1—C6—C7	179.26 (15)	C13—C14—C15—C16	177.79 (16)
F2—C5—C6—C1	177.68 (13)	F3—C10—C15—C14	179.69 (14)
C4—C5—C6—C1	−0.3 (2)	C11—C10—C15—C14	0.5 (2)
F2—C5—C6—C7	−0.2 (2)	F3—C10—C15—C16	1.7 (2)
C4—C5—C6—C7	−178.22 (15)	C11—C10—C15—C16	−177.44 (16)
C8—N1—C7—C6	179.47 (14)	C17—N2—C16—C15	178.58 (14)
C1—C6—C7—N1	166.67 (15)	C14—C15—C16—N2	−179.69 (15)
C5—C6—C7—N1	−15.5 (2)	C10—C15—C16—N2	−1.8 (3)
C7—N1—C8—C9	119.39 (16)	C16—N2—C17—C18	118.07 (16)
N1—C8—C9—C9^i^	−66.7 (2)	N2—C17—C18—C18^ii^	−66.8 (2)

Symmetry codes: (i) −*x*, −*y*+1, −*z*+1; (ii) −*x*, −*y*, −*z*+2.

### Hydrogen-bond geometry (Å, °)

**Table:**

*D*—H···*A*	*D*—H	H···*A*	*D*···*A*	*D*—H···*A*
C3—H3···F3^iii^	0.95	2.43	3.137 (2)	131
C7—H7···F1^iv^	0.95	2.54	3.378 (2)	148
C12—H12···F2	0.95	2.42	3.192 (2)	138

Symmetry codes: (iii) *x*+1, *y*+1, *z*; (iv) −*x*, −*y*+2, −*z*+1.
